# A Study on the Relationship between the Dynamic Behaviors of the Leader and Group Performance during Creativity

**DOI:** 10.3390/jintelligence10040087

**Published:** 2022-10-13

**Authors:** Qingbai Zhao, Ying Li, Songqing Li, Zheng Liang, Shi Chen, Riman Ga, Quanlei Yu, Zhijin Zhou

**Affiliations:** 1 Key Laboratory of Adolescent Cyberpsychology and Behavior (CCNU), Ministry of Education, Central China Normal University, Wuhan 430079, China; 2 Key Laboratory of Human Development and Mental Health of Hubei Province, School of Psychology, Central China Normal University, Wuhan 430079, China; 3 School of Preschool Education, Changsha Normal University, Changsha 410100, China; 4 College of Electronic Engineering, Naval University of Engineering, Wuhan 430034, China; 5 College of Humanities, Hubei University of Chinese Medicine, Wuhan 430065, China

**Keywords:** group creativity, emergent leader, elected leader, interpersonal interaction, dynamic behaviors

## Abstract

The leader is considered to play key roles such as organization or management in promoting group creativity. Previous studies focused more on the psychological and behavioral characteristics rather than on the dynamic behaviors of leaders in group activity. In this study, two experiments were conducted to respectively explore the effects of emergent and elected leaders’ problem-solving related utterances and turn-taking in conversation on group creativity. The results of Experiment 1 showed that, for emergent leaders, none of the utterances about problem solving of leaders was different from that of followers and leaders’ utterances about retrospective summary were positively related to the appropriateness of group creativity; meanwhile, the frequency of turns of leaders was higher than that of followers and was positively related with the appropriateness of group creativity. The results of Experiment 2 showed that, for elected leaders, the utterances about problem analysis, strategy planning, control and reflection, and retrospective summary of leaders were more than that of followers and leaders’ utterances about viewpoint generation were positively related to both novelty and appropriateness, while the frequency of turns of leaders was neither different from followers nor related to the novelty and appropriateness. This study focused on the dynamic behaviors of leaders in interpersonal interaction and revealed the role of leaders in group creativity.

## 1. Introduction

Creativity is the process of generating products or ideas that are novel, appropriate, and valuable (Hennessey and Amabile 2010). Creating in groups has become an important form of learning and working (Stachowski et al. 2009). In the process of group creativity or problem solving, members have different roles, and their responsibilities and contributions in the group are not equal (Baker 2002). Meanwhile, the leader in the group has been considered to play a key role in promoting organizational performance (Bass and Stogdill 1981, 1990; Huang et al. 2016; Kock et al. 2019; Lorinkova et al. 2013; Veestraeten et al. 2021). We focus on the impact of leader on group performance in the process of group creativity.

Group creativity is a process in which members collaborate to generate novel and appropriate ideas about products, services, and processes at the group level (Amabile 1988; Shin and Zhou 2007). The process of group creativity is similar to the individual creative thinking process, in which creative products or ideas are generated through the stages of preparation, creative focus, divergent thinking, incubation, and convergent thinking (Leonard-Barton and Swap 1999). Meanwhile, the process of group creativity is also a distributed creative process (Sawyer and DeZutter 2009), in which members within a group generate creative ideas or thoughts based on their respective knowledge systems and form a consensus on creative answers through interpersonal interactions such as collaboration. It could be inferred that group creativity is the creative outcome of group interaction.

Some theoretical models of group creativity have explored the cognitive process of group creativity. For example, the cognitive model of group creativity (SIAM, Search for Ideas in Associative Memory) focused on the process of repeated search for ideas in associative memory, arguing that individuals use internal cues to search for relevant information from long-term memory to form new ideas, and group members’ attention to others’ perspectives can also serve as external cues to facilitate the generation of their respective ideas, while group interactions can facilitate or hinder this process (Nijstad et al. 2003; Nijstad and Levine 2007; Nijstad and Stroebe 2006). This model pays attention to the group cognitive process by which members’ ideas are generated and emphasizes that the interaction between members is more about the attention and integration of other people’s ideas. Paulus proposed the cognition-social-motivation (CSM) model of collaborative creativity based on existing research, which suggested that the group creativity process includes cognitive processes, social processes, motivational processes, and their mutual influence (Paulus et al. 2012). The cognitive processes are concerned with the processes of solutions generation which can be generated by searching from long-term memory, attending to others’ ideas, and combining/elaborating on previously generated ideas and others’ ideas. The motivational processes refer to using internal motivators and external motivators to set and maintain a high level of motivation and also includes reducing group motivational losses. The social processes include sharing generated ideas, exchanging information/collaborative problem-solving, discussing varied viewpoints/minority dissent, engaging in social comparison, and managing conflict and reflexivity. This model focuses on the influence of variables such as group member variables, group structure, group climate, external demands in the process of interpersonal cognitive-social interaction in group creativity, and team innovation.

In fact, group creativity is a special form of group problem solving. Not all problems require creative solutions, and not all cognitive processes would produce creative results (Brophy 1998). Creativity will occur when there is a need for an innovative solution to a general problem and can only be solved through cognitive processes. West (2002) also proposed that group creativity is a process by which members as a whole engage in problem solving and generate novel and appropriate products. In order to understand the macrocognition in groups, Fiore synthesized multidisciplinary theoretical and empirical literature related to knowledge work in teams, and proposed that the stages of group problem solving include four collaboration phases: knowledge construction, team problem model, team consensus, and evaluation/revision (Fiore et al. 2010). 

Meanwhile, the group process is particularly important in group problem solving. Marks et al. (2001) suggests that this process refers to the interdependent acts of group members that convert inputs into outcomes through cognitive, verbal, and behavioral activities, and these acts are directed by organizing taskwork to achieve collective goals. It can be reasoned that the group process in group problem solving encompasses both the process of problem solving and interpersonal interaction of group members. Some researchers proposed to explore the problem solving process and interpersonal interaction process from the perspective of the utterance content and characteristics of the interaction (Bales and Strodtbeck 1951). For example, Hirokawa (1982) argued that the utterances of group members are functional, and members’ statements and inquiries are the main ways of group discussions. He proposed a function-oriented interaction analysis system for group interaction data, which divided group interactions in the problem-solving process into four task-achievement functions: establishment of operating procedures, analysis of the group problem, generation of alternative suggestions, and evaluation of alternative suggestions, through the introduction, restatement, development, substantiation, modification, agreement, and other subcategories of verbal utterances to accomplish the problem-solving process or group decision making. Simon (2002) developed a group interaction observation system from the perspective of interaction effectiveness, and proposed that the complex problem-solving processes include goal clarification, process clarification, problem analysis, problem solving, and process control. Interactive utterances in each process include statement, inquiry, argument, suggestion, and so on. The creative problem solving used in this study is also a complex problem solving, so the interactive process of it could also be referred to this model.

In the interpersonal interaction during the discussion, the leaders’ behavior will affect the outcome of group creativity. Zaccaro et al. proposed that effective leadership is the most critical factor in the success of organizational teams (Zaccaro et al. 2001). As the core member of the group, the leader organizes and manages the discussion during the problem-solving process to move the discussion forward and ultimately achieve the group goals (Lanaj and Hollenbeck 2015).

There is ample research that has explored the relationship between leadership and group creativity (Hughes et al. 2018), including the effects of personal traits of leaders, leadership style or leadership behaviors, and the leader–follower relationships on creativity. In terms of the personal traits of leaders, related research has focused on the influences of the traits such as creativity level, willingness to take risks, and psychological empowerment on group creativity. For example, Li and Yue proposed that the leader’s level of creativity is a core component of effective leadership, and was positively related to group creativity, and moderated by leader empowerment and task complexity (Li and Yue 2019). Duan et al. examined the relationship between ethical leadership and employee creativity in Chinese businesses and found that leaders’ psychological empowerment and willingness to take risks mediated the relationship between ethical leadership and employee creativity (Duan et al. 2018). Hu et al. analyzed the data related to leadership humility and group creativity in 72 groups and found a significant positive correlation between leadership humility characteristics and team information sharing (Hu et al. 2018). Related research on leadership style or leadership behaviors has also found that shared leadership, transformational leadership, and leadership strategies all have influences on group creativity. For example, He et al. examined the way of shared leadership and formally appointed leaders’ transformational leadership in cultivating group creativity in two studies, and found that shared leadership improved group creativity by enhancing members’ creative self-efficacy and individual creativity (He et al. 2020). Javed et al. explored the relationship between ethical leadership and employee creativity and found the moderating role of leadership trust and openness to experience (Javed et al. 2018). Koh et al. explored the relationship between transformational leadership and creativity through a meta-analytic review and found that there was a stronger relationship between transformational leadership, creativity, and cultural values among employees in Asian countries compared to Western countries (Koh et al. 2019). Gu et al. revealed whether and how shared leadership affects different levels of creativity through a multilevel motivational mechanism (Gu et al. 2022). Lee et al. explored the relationship between leadership strategies, staff’s creativity, and group performance (Lee et al. 2021). He found that brainstorming and reflection were helpful for the leaders to seek disruptive change and implement the ideas. Other studies have also partially explored the impact of leader–follower relationships on group creativity. For example, Liu et al. found that follower creativity can be stimulated when there are different risk orientations between leaders and followers, and that leader authority openness moderates the indirect effect of leader–follower risk orientation incongruence on creativity through follower–experienced intellectual stimulation (Liu et al. 2021). Related research on leader–follower relationships characterized by LMX quality have found that leaders may stimulate creative and innovative performance by providing followers with high levels of autonomy and discretion (Pan et al. 2015), allocating needed resources (e.g., Gu et al. 2015), and building followers’ confidence (Liao et al. 2010).

Most of the above studies focus on leaders’ influence on employee behavior and group creative performance from the perspective of psychological and behavioral types, and less on the dynamic behaviors of leaders in the group creative process. Different from those studies, by reviewing the cognitive processes underlying creative problem solving, Reiter-Palmon et al. suggested the avenues to promote creative outcomes through which organizational leaders can facilitate these processes, based on previous relevant research (Reiter-Palmon et al. 2008). During the problem construction stage, leaders may facilitate this process by drawing the subordinates’ attention to the importance of problem construction and thinking about the problem from multiple perspectives. During the information search and encoding stage, leaders may facilitate this process by managing the acquisition, sharing, and distributing of knowledge and information among their employees. During the solution generation stage, leaders can influence the results of this process by the instructions they provide to subordinates. During the idea evaluation stage, leaders can complete the process by articulating criteria for evaluating solutions and creating a culture of open communication and mutual trust. Although this study illustrates possible ways in which leaders can facilitate creative performance in various stages of problem solving from a cognitive perspective, the authors do not take into account the fact that the leader, as a member of the group, is also a participant in the problem-solving process, as he or she played an organizing and managerial role in this study. Leaders are not only organizers of groups but also members, so the leadership process should function in the context of shared group membership (Van Knippenberg and Hogg 2003). At the same time, in the current highly competitive and dynamic environment, there is an increasing emphasis on knowledge-based team structures (Bligh et al. 2006), which spread leadership across multiple group members rather than concentrating it on one individual (Zhu et al. 2018). Therefore, it is worth exploring further how the dual identity of problem solver and organizer of leader in knowledge-based groups can play a role in creative problem solving.

In summary, leaders play an important role in group creativity, but previous related studies have focused more on leaders in organizational management and less on leaders’ identity as problem solvers; and they have explored the influence of leaders’ psychological and behavioral characteristics on group creativity more from a static perspective, and less on their leadership behaviors in the process of group problem solving. Therefore, this study focuses on both the organizer and problem-solver identities of leaders and explores the role of leaders in group creativity from a dynamic perspective.

Yukl et al. proposed that the most prominent behavior of the leader is the control of team interaction, which guides and facilitates the problem-solving process to achieve group goals, and suggested that this control mainly includes task-oriented behaviors and relations-oriented behaviors (Yukl et al. 2002). In terms of task-oriented behaviors, the leader facilitated problem solving by planning short-term activities, clarifying task objectives and role expectations, and monitoring operations and performance. In terms of relations-oriented behaviors, leaders interact with members through cognitive, verbal, and action interactions, which provide support and encouragement to group members, recognize members’ achievements and contributions, develop members’ skills and confidence, consult with members when making decisions, and empower members to take initiative in problem solving. At the same time, in the process of group interaction, members come up with creative ideas and perspectives based on their different knowledge systems, and the leader, as a group member, is also a generator of novel ideas (Gerpott et al. 2019). Compared to other members, the leader is generally expected to have high level of creative thinking skills and is able to take the lead in developing novel and appropriate ideas or problem solutions (Mumford and Licuanan 2004). In general, through task-oriented behaviors, leaders guide the problem-solving process and provide technical and methodological guidance to members to facilitate the completion of creative tasks. Through relations-oriented behaviors, leaders motivate members, coordinate and guide members to achieve the goal, thereby enhancing group creative performance. Therefore, we hypothesized that the leader’s level of involvement in the group problem-solving process and interpersonal involvement with followers as problem solvers and organizers would have an impact on group creative performance.

Language is a very important means of communication in face-to-face conversation (Roberts et al. 2015). During the process of group interaction, on the one hand, utterance serves as an important information carrier through which members can exchange their ideas, communicate with each other, facilitate problem solving, and reach consensus. On the other hand, the communication behaviors such as turn-taking and interjections can also convey information to others (Jiang et al. 2015). By analyzing the utterance contents and the turn-taking during the process of group interaction, we could reveal the mechanism of the leaders’ participation in group problem solving that affects group performance from a dynamic perspective. In terms of the utterance related to problem-solving, we hypothesize that the quantity of leaders’ utterance reflects their involvement in the problem-solving process, which ultimately affects the performance of the group, but the influence of each stage is still needs to be further explored. In terms of verbal communication behavior of the turn-taking, we hypothesize that the frequency of leaders’ turn reflects their interactions with followers, which will ultimately also affect the final performance of the group.

It is worth mentioning that the two ways of leaders’ effects do not exactly coincide in different types of groups. In groups whose leader is formally appointed, the content of the leader’s authority is clear, and there is a clear division of hierarchical status among members (Moreland 2010). In contrast, in project groups of organizations or in learning groups of universities and research institutions, groups members are de-stratified, with relatively equal relationships and blurred role boundaries (Denison et al. 1996), and leaders engage in more horizontal communication and exchange, retaining their identity as knowledge members (Faraj and Yan 2009), and focusing on the problem-solving process. The latter focus on the learning, exchanging, and sharing of knowledge can better cope with complex cognitive tasks and demonstrate high level of creativity (Oborn and Dawson 2010). At the same time, the role of leaders in group interactions is not entirely consistent across groups. Leaders in project or learning groups have more task-based interactions with their members, so studying leaders in such groups is a better way to explore their role in the group’s creative process. In addition, leaders of groups can be spontaneously generated from members during the process of task completion, such as the emergent leaders in leaderless groups being spontaneously generated as the task progresses (Jiang et al. 2015). The emergent leader guides the group’s problem-solving thinking through interpersonal communication with members, with whom there is no clear leader–follower relationship and individuals in the group are on equal social role status. Alternatively, leaders can also be appointed by members through free election before the task (Elgie et al. 1988). The elected leader serves as the group project manager, performing a leadership function and forming a clear leader–follower relationship with the members. Therefore, this study focused on leadership behavior in group interaction, and adopted two experiments to explore the relationship between the dynamic behavior of emergent and elected leaders and group creativity.

To explore the role of leader’s behavior during the interaction with followers in group creativity, this study adopts the interaction process analysis method and conversation sequence analysis method to analyze the group interaction process from the perspective of utterance contents and dialogue directions. 

The “Interaction Process Analysis” observation system developed by (Bales and Strodtbeck 1951) provides a research method basis for group research and is highly praised by small group research (McGrath 1984). On this basis, Simon (2002) developed another group interaction observation system from the perspective of group effectiveness which can analyze the semantic content and directly relate to the group task performance. The system developed by Simon divides the utterance during problem solving into five broad categories: goal clarification, process clarification, problem analysis, problem solving, and process control. Combing the dynamic characteristics of group decision making and the advantage and disadvantages of each observation coding system mentioned above, He and Tjitra (2009) put forward the Multiple Group Process Coding System (MGPC) and divide the problem solving process into 5 main categories: goal clarification, problem analysis, strategic planning, problem solving, and process control and reflection. Based on this coding system, the research could analyze the dynamic process of group complex problem solving, and further evaluate the interactive process of task-oriented and interpersonal-oriented. It also could be used to explore the contribution of each member in the process and the mechanism of the influence on task performance. The interaction process analysis method adopted in this study combines MGPC and the characteristics of this study and was used to analyze the attributes of utterance content in group problem solving. The leader communicates and interacts with followers through utterances to facilitate creative problem solving. Therefore, using the interaction process analysis method to analyze utterances during group discussions could better reveal the role of the leader in group creativity. 

In addition to the content of the utterance, turn-taking is also a very important behavior of communication which included verbal communications such as turn-taking and interjection and nonverbal communications such as facial expressions and sign gestures (Holler et al. 2018; Jiang et al. 2015). The analysis of turn-taking can reflect interpersonal relationships (Ravenet et al. 2015). Jiang et al. (2015) analyzed the frequency and quality of communications through encoding these communication behaviors. In this study, the conversation sequence analysis method was adopted to compare the characteristics of turn-taking of the leader and the follower. By analyzing the turn-taking between them during group discussions, it is possible to reveal the ‘black box’ in which leaders facilitate group consensus by driving the discussion process, and thus explore the role of relations-oriented leader behaviors in group creativity.

## 2. Experiment 1

The aim was to investigate the role of emergent leaders in group creativity by analyzing the utterances of emergent leaders about problem solving and their turn-taking in interpersonal interactions during the process of group creative problem solving.

### 2.1. Methods

#### 2.1.1. Participants

The sample size was estimated in advance through a power analysis conducted using G*Power 3 (Faul et al. 2009) with power (1 − β) set at 0.8, α = 0.05 (2-tailed), and a modest effect size of f = 0.25. The analysis yielded the required sample size of 28. Thus, we recruited 102 college students (90 females, mean age ± standard deviation (SD): 20.87 ± 1.74 years, range 18–27 years) to participate in this experiment, with every 3 students of the same sex in one team, forming a total of 34 teams, and the three members in the same team did not know each other before the experiment. Among the 34 groups, the members of one group did not communicate with each other during the experiment, which led to the failure of the group task. The members of three groups were off the task topic several times during the discussion. Thus, 30 groups (26 female groups and 4 male groups) remained after eliminating these 4 groups. All participants were right-handed native Chinese speakers. All participants signed informed consent forms before the experiment, and all received some payment after the experiment.

#### 2.1.2. Experimental Design

The experiment adopted a one-way experimental design. The independent variable was the role of the members, who were sequentially classified into three categories: leader (L), follower 1 (F_1_), and follower 2 (F_2_), based on the self-assessment leadership scores after the experiment. The dependent variables included the quantity of utterances, the frequency of turns, and the index of group creativity.

For the dependent quantity of utterance, we subdivided it into 6 categories according to the meaning of each sentence: the quantity of utterances about goal clarification (GC), the quantity of utterances about problem analysis (PA), the quantity of utterances about strategy planning (SP), the quantity of utterances about viewpoint generation (VG), the quantity of utterances about control and reflection (CR), and the quantity of utterances about retrospective summary (RS).

For the frequency of turns, we also subdivided it into 6 categories according to the direction of each turn: the frequency of turns of leader (L←F_1_) and follower 1 (F_1_←L) in the conversation of the leader and follower 1, the frequency of turns of leader (L←F_2_) and follower 2 (F_2_←L) in the conversation of the leader and follower 2, and the frequency of turns of follower 1 (F_1_←F_2_) and follower 2 (F_2_←F_1_) in the conversation of follower 1 and follower 2.

For the index of group creativity, it also contains novelty and appropriateness of group performance evaluated by professionals.

#### 2.1.3. Subjective Measurement

- Assessment of the interpersonal communication ability: The Interpersonal Communication Ability Questionnaire developed by (Zhang et al. 2004) was adopted to measure the individuals’ interpersonal communication ability. This questionnaire consists of three dimensions: communication skills, communication perceptions, and communication tendencies, with a total of 15 items. The questionnaire uses a 5-point Likert scale (valence: 1 = never, 5 = always), and the mean value of the three dimensions was taken as the level of individual interpersonal communication ability, and a higher score represents a stronger interpersonal communication ability. Cronbach’s α was 0.86 in this study.
- Assessment of the extraverted personality: The extraversion dimension of The Big Five Personality Questionnaire was used to measure the extraverted personality traits of the participants, with 13 items, using a 5-point Likert scale (valence: 1 = never, 5 = always). The mean value of all items was taken as the individual extraversion score, with higher scores indicating higher level of extraversion. Cronbach’s α was 0.70 in this study.
- Assessment of the cooperative preference: We used the Chinese version of the Group Preference Scale (GPS) developed by Larey and Paulus (1999) to measure the individuals’ cooperative preference. The scale has 10 items and uses a 5-point Likert scale (valence: 1 = not at all, 5 = very much). The mean value of 10 items was taken to obtain the GPS score, with higher scores indicating higher level of cooperative preference. In the present study, Cronbach’s α was 0.86.
- Assessment of the leadership: Based on the Leadership Emergence Questionnaire developed by Lanaj and Hollenbeck (2015), we used a self-assessment method by asking the participants to indicate the degree to which they emerged as a leader via items that were descriptive of whether they led or did not lead. The scale has 5 items and is rated on a 7-point Likert scale (valence: 1 = very low; 7 = very high), the mean value of all items was taken as the individual leadership level. In the present study, Cronbach’s α was 0.92.

Assessment of the creative ability: The AUT task was used to measure the participants’ creative ability by asking them to come up with as many special uses for a paper box as possible within 5 min (Stoltzfus et al. 2011). All answers were rated on a 5-point Likert scale of creativity (valence: 1 = very low; 5 = very high) by five professionals, and the mean value of the answers was used as the individual creative ability score, with higher scores indicating higher creative ability. In the present study, Cronbach’s α was 0.84.

#### 2.1.4. Experimental Procedure

Before the experiment began, the participants filled out their personal information and completed the pre-test assessment including their interpersonal communication ability, extraverted personality, cooperative preference, and creative ability.

**Figure 1:**
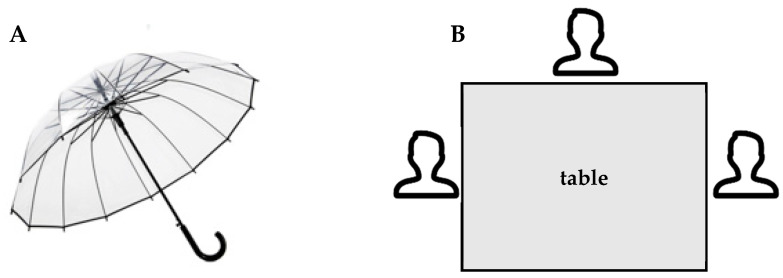
Umbrella prototype and seating arrangement. (**A**) the umbrella prototype, which was presented to the participants during the instruction process, so that the group members had a consistent umbrella prototype, and the prototype picture was retrieved before the formal discussion. (**B**) The seating arrangement, in which the three members freely chose seats according to the seating arrangement and two cameras were used to record the discussion.

Afterward, three members randomly chose seats (the seating arrangement is shown in Figure 1B) and then collaborated to complete the “Creative Product Improvement Task”. This task is an item on the Torrance Tests of Creative Thinking (TTCT), which is typically used to measure individual creativity (Torrance 1984). To make the task relevant to daily life, we replaced the elephant, the object of the original creative product improvement task, by an umbrella. The instructions and requirements for this task were as follows: “The creative product improvement task requires everyone to work together to solve a problem. An umbrella is indispensable in life! The umbrella in the picture (see Figure 1A) is very ordinary. Please cooperate and discuss how to improve this umbrella into a novel umbrella. You need to form a complete creative improvement plan through discussion, and it should be novel, appropriate and fine”. The whole discussing process of group problem solving was recorded by two cameras placed in the corner of the lab.

Finally, all members completed the assessment of the leadership and rated the difficulty of the group task on a 5-point scale (valence: 1 = very easy; 5 = very difficult).

#### 2.1.5. Data Encoding

Throughout the experiment, we video-recorded the discussion process of group problem solving. Undergraduate psychology students were recruited to transcribe the conversation about group problem solving sentence by sentence. The direction of turns, the initiator, and the receiver were also labeled. Appropriate training was provided before transcription. After the transcription was completed, two professionals coded the utterance content of group problem solving and the sequence of the turn-taking.

**Figure 2:**
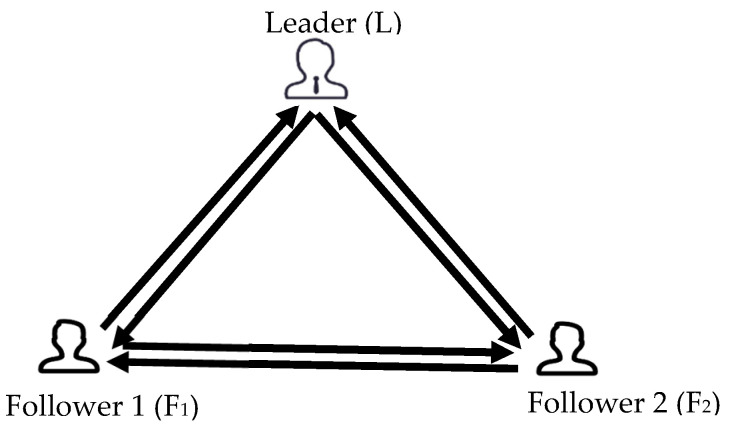
Schematic diagram of the turn-taking of the conversion.

- Utterance content coding: We conducted a pre-experiment whose experimental procedure and transcript were aligned with the formal experiment to verify the consistency of the group problem-solving process between this study and the group problem-solving model. In the pre-experiment, 18 university students were divided into 6 groups to solve the group creative problem solving and the entire discussion process was recorded. By combining the Multiple Group process Coding System (MGPC) proposed by (He and Tjitra 2009) and the expert analysis, it was concluded that the problem-solving process of the group is that the group clarifies the goals of the task at first, then analyzes the problem to be solved. After that, the group would develop a strategic plan and then propose ideas for problem solving, while the control and reflection on the discussion process are carried out throughout the entire discussion. Thus, based on these results of the pre-experiment, we clarified the utterance content about problem solving into six categories: goal clarification (GC), problem analysis (PA), strategy planning (SP), viewpoint generation (VG), control and reflection (CR), and retrospective summary (RS). The meaning and examples of each category are shown in Table 1. Two professional coders who were not involved in the evaluations of group creative performance coded the utterance content into different categories sentence by sentence. These two coders first pre-coded two conversations, and their consistency coefficients Cronbach’s α were 0.89 and 0.84, then they coded all the conversations separately.
- Conversation sequence coding: Referred to the communication behaviors coding method adopted by (Jiang et al. 2015), two professional coders coded the directions of the turn-taking as well as the initiator and receiver of every turn. According to the directions between different roles, the conversations between members were classified into six categories (shown in Figure 2): the turns of the leader take from the follower 1 (L←F_1_), the turns of the leader take from the follower 2 (L←F_2_), the turns of the follower 1 take from the leader (F_1_←L), the turns of the follower 2 take from the leader (F_2_←L), the turns of the follower 2 take from the follower 1 (F_2_←F_1_), the turns of the follower 1 take from the follower 2 (F_1_←F_2_). The frequency of turns of different roles was calculated.
- The assessment of group creative performance: Five graduate students used the consensus assessment technique (Amabile 1983) to evaluate the novelty and appropriateness of the problem solution on a Likert scale ranging from 1 (not at all) to 7 (extremely). The rater agreement coefficients, i.e., Cronbach’s α, of the novelty and appropriateness, were 0.82 and 0.75, respectively. The mean scores of the corresponding scores of the five raters were used as the novelty and appropriateness scores of the group creative performance.

### 2.2. Results

#### 2.2.1. Individual Differences between Roles

Before analyzing the dynamic behaviors of leaders and their relationship to group performance, we tested whether the individual characteristics differed across roles. A series of repeated measures ANOVA analysis of variance was used to compare the leadership, interpersonal communication ability, extraverted personality, cooperative preference, and creative ability of different roles within the group. The results about the leadership showed that there were significant differences in leadership among roles (F (2, 29) = 69.95, *p* < 0.001, η^2^_partial_ = 0.71, 95%CI [0.67, 0.88]). Post-hoc multiple comparisons found that the leadership of the leader (M = 5.21, SD = 0.67) was significantly higher than that of follower 1 (M = 4.45, SD = 0.52, *p* < 0.001) and follower 2 (M = 3.43, SD = 0.71, *p* < 0.001), the leadership of follower 1 was significantly higher than that of follower 2 (*p* < 0.001). Meanwhile, there were also significant differences in interpersonal communication ability between roles (F (2, 29) = 3.22, *p* = 0.047, η^2^_partial_ = 0.10, 95%CI [0.00, 0.38]), post-hoc multiple comparisons found that the interpersonal communication ability of leaders (M = 3.58, SD = 0.38) is significantly higher than those of followers 2 (M = 3.36, SD = 0.49, *p* = 0.031), there were no significant differences between leader and follower 1 (M = 3.54, SD = 0.33) and between the two followers. However, there was no significant difference between roles on the extraversion, cooperative preference and creative ability (extroversion: F (2, 29) = 2.03, *p* = 0.141; cooperative preference: F (2, 29) = 0.28, *p* = 0.754; creative ability: F (2, 29) = 0.84, *p* = 0.438). We also compared the task difficulty assessed by members using repeated-measures ANOVA analysis, and the results showed that there were significant differences in the task difficulty scores among roles (F (2, 29) = 7.51, *p* = 0.001, η^2^_partial_ = 0.21, 95%CI [0.06, 0.53]). Post-hoc multiple comparisons found that the difficulty assessed by the leader (M = 1.37, SD = 0.10) was significantly lower than that by follower 2 (M = 2.23, SD = 0.20, *p* = 0.001), while there was no difference between the leader and the follower 1 (M = 1.60, SD = 0.17, *p* = 0.229), and the difficulty assessed by follower 1 was significantly lower than by follower 2 (*p* = 0.028).

#### 2.2.2. Behavioral Characteristics of the Emergent Leader in the Process of Group Creative Problem Solving

A series of repeated measures ANOVA analysis were used to compare differences in the quantity of different roles’ utterances during creative problem solving in order to explore the problem-solving behavior of emergent leaders. The results revealed that there was no significant difference in the total quantity of utterances during problem solving among leaders, follower 1 and follower 2 (F (2, 29) = 0.45, *p* = 0.638). At the same time, there was no significant difference between the quantity of the three roles’ utterances about goal clarification, problem analysis, strategy planning, viewpoint generation, control and reflection, and retrospective summary (GC: F (2, 29) = 0.20, *p* = 0.819; PA: F (2, 29) = 0.52, *p* = 0.595; SP: F (2, 29) = 0.94, *p* = 0.398; VG: F (2, 29) = 0.03, *p* = 0.969; CR: F (2, 29) = 0.44, *p* = 0.648; RS: F (2, 29) = 1.89, *p* = 0.160).

In addition, we also calculated Pearson correlation coefficients between the quantity of different utterances of emergent leaders and every indicator of group creativity to reveal the role of emergent leaders in group creative performance. The results are shown in Table 2, the total quantity of the leader’s utterances was neither correlated with novelty nor with appropriateness (*p* > 0.05). Meanwhile, the quantity of leader’s utterances about retrospective summary was positively correlated with the appropriateness significantly (*r* = 0.38, *p* = 0.038), while there was no significant correlation between the quantity of utterances about goal clarification, problem analysis, strategy planning, viewpoint generation, control and reflection, and every indicator of group creative performance (*p* > 0.05).

#### 2.2.3. The Characteristics of Turn-Taking of Emergent Leaders in Interpersonal Interactions

The turn-taking in the conversation between leaders and followers is also an important communication behavior; analyzing the characteristics of turn-taking of emergent leaders is another way to reveal the role of emergent leaders in group creative performance. Considering the difference in the total quantity of turns in each group, we used the ratio of the quantity of turns in each direction to the total number of turns as the frequency of turns in different conversation for the subsequent analysis. We used the sum of the frequency of turns of L←F_1_ and L←F_2_, the sum of the frequency of turns of F_1_←L and F_1_←F_2_, and the sum of the frequency of turns of F_2_←F_1_ and F_2_←L as the total frequency of turns of the leaders, followers 1, and followers 2, respectively. Repeated measures ANOVA analysis was used to compare the differences in the frequency of turns between roles; meanwhile, a series of paired samples *t*-tests were used to compare the differences between the frequency of turns of leaders and followers in different conversations.

Repeated measures ANOVA analysis revealed that there were significant differences in the total frequency of turns across roles (F (2, 28) = 6.13, *p* = 0.004, η^2^_partial_ = 0.18, 95%CI [0.03, 0.48]). Post-hoc multiple comparisons indicated that the total frequency of turns of the leader (M = 0.43, SD = 0.17) was significantly higher than that of follower 1 (M = 0.27, SD = 0.12, *p* = 0.002) and follower 2 (M = 0.30, SD = 0.16, *p* = 0.031), while there was no significant difference between the two followers (*p* = 0.432). Paired-samples *t*-tests were used to compare the differences in the frequency of turns between L←F_1_ and F_1_←L, L←F_2_ and F_2_←L, L←F_1_ and L←F_2_, and F_1_←L and F_2_←L, respectively. We found that the frequency of turns of L←F_1_ (M = 0.22, SD = 0.09) was significantly higher than F_1_←L (M = 0.14, SD = 0.06) (t (29) = 3.43, *p* = 0.002, Cohen’s d = 0.63, 95%CI [0.23, 1.01]); the frequency of turns of L←F_2_ (M = 0.21, SD = 0.09) was significantly higher than F_2_←L (M = 0.15, SD = 0.08) (t (29) = 2.06, *p* = 0.048, Cohen’ s d = 0.38, 95%CI [0.003, 0.74]); there were no differences between the frequency of turns of L←F_1_ and L←F_2_ (t (29) = 0.89, *p* = 0.38), and between F_1_←L and F_2_←L (t (29) = 0.93, *p* = 0.36).

In addition, we also calculated the Pearson correlation coefficients between the frequency of turns of L←F_1_, L←F_2_, F_1_←L, F_2_←L and every indicator of group creativity. The results were shown in Table 3, the frequency of turns of L←F_1_ and L←F_2_ were both positively correlated with the appropriateness of the group creativity significantly (*r*_L←F1_ = 0.37, *p* = 0.045; *r*_L←F2_ = 0.41, *p* = 0.025).

### 2.3. Discussion

Leaderless group discussion is often used to evaluate individual leadership potential and traits. Although there is no pre-designated leader, most groups will eventually spontaneously form the “leader” of the group through discussion. In this experiment, three participants who did not know each other formed a temporary group and worked together to complete the creativity task. By analyzing the dynamic behaviors of emergent leaders in problem solving and their relationship with group creativity during group discussions, the mechanism of emergent leadership in group creativity was revealed.

In this experiment, the members were classified into the emergent leader, follower 1, and follower 2 based on their post-hoc self-assessment of leadership. A meta-analysis about leadership behaviors and group performance found that the relationship between leadership behaviors and group performance was stronger when leadership behaviors were assessed by the leaders themselves rather than by others (Ceri-Booms et al. 2017). Therefore, we used the self-assessment method rather than the other-assessment method to distinguish between the different roles in this study. Then, by comparing the differences between the interpersonal communication ability, extroverted personality, and cooperative preferences among the three roles, we found that there are no significant differences between them except that the interpersonal communication ability of the emergent leader was significantly higher than that of follower 2. These results suggested that individual differences among members are not significant. In this experiment, the group was formed temporarily, and the interaction between members was more about the task, so the stable personality differences such as extraversion and cooperative preferences may not be fully exposed in such unfamiliar situations. However, interpersonal communication ability is important precisely because the interactions among members are task-oriented. Individuals with strong interpersonal communication abilities tend to have more interpersonal interactions (Leaper 1987). Meanwhile, they also have stronger empathy and are able to use transpersonal thinking (Riggio et al. 2003; Riggio and Friedman 1982). It could be inferred that the individuals who exhibit strong interpersonal communication ability have greater influence on others during interactions and emerge as the leader.

Furthermore, in this experiment, by comparing the quantity of utterances about problem solving of emergent leaders and followers, we found that there was no significant difference between emergent leaders and followers in the total quantity of utterances, and the quantity of utterances about goal clarity, problem analysis, strategy planning, idea generation, control and reflection, and retrospective summary also did not differ significantly from that of the followers, but the quantity of utterance about retrospective summary of the emergent leader was positively related to the appropriateness of group creativity. Meanwhile, by comparing the frequency of turns of emergent leaders and followers during turn-taking, we found that emergent leader was significantly higher than two followers in terms of the total frequency of turns. As to conversational reciprocity, it was shown that emergent leaders take turns more frequently compared to the opposite direction, and the frequency of the emergent leader was also related to the appropriateness of group creativity.

The effects of the leader in the group cannot be ignored. Even though in the leaderless group discussion, a leader will emerge through interpersonal interaction, in order to be more organized and more effective, the group will appoint a leader before the task, and the appointed leader will lead members to complete the task. Whether the mechanism of the role of pre-designated leaders in group creativity is the same as that of emergent leaders needs to be further explored. Therefore, in Experiment 2 of this study, we explored the mechanism of the role of elected leaders in group creativity by identifying leaders in advance of the election task.

## 3. Experiment 2

According to social role theory, individuals who are assigned a role will exhibit behaviors that are consistent with what is expected of him/her in that role (Van Lange et al. 2011). When an individual is assigned as a leader, he or she will exhibit behaviors such as organizing and managing the group discussion process, influencing individuals or organizations through personal behaviors, and ultimately contributing to the achievement of group goals. This experiment explored the role of elected leaders in group creativity by analyzing the utterance of elected leaders about problem solving and their turn-taking in interpersonal interaction during the process of group creative problem solving.

### 3.1. Methods

#### 3.1.1. Participants

Sample size was estimated in advance through a power analysis conducted using G*Power 3 (Faul et al. 2009) with power (1 − β) set at 0.8, α = 0.05 (2-tailed), and a modest effect size of f = 0.25. The analysis yielded the required sample size of 28. Thus, we recruited 102 college students (87 females; mean age standard deviation (SD) 20.28 ± 1.61 years; rang 18–25 years) to participate in this experiment, with every 3 students of the same sex in one group, forming a total of 34 groups, and the three members in a group did not know each other before the experiment. The members of two groups did not communicate with each other during the experiment, which led to the failure of the group task, and two other groups had incomplete data due to the interruption of video recording. After excluding these four groups, data from the remaining 30 groups (25 female groups and 5 male groups) were included in the subsequent analysis. All participants were right-handed native Chinese speakers. All participants signed informed consent forms before the experiment, and all received some payment after the experiment.

#### 3.1.2. Experimental Design

The experiment adopted a one-way experimental design. The independent variable was the role of the members including the leader, follower 1, and follower 2. The leader was elected by the members of the group through the leader election task, while the role of the follower 1 and follower 2 was then decided based on the self-assessment leadership scores after the experiment. The dependent variables were the same as in Experiment 1.

#### 3.1.3. Subjective Measurement

Subjective measurements in Experiment 2 including the assessment of the interpersonal communication ability, extraverted personality, cooperative preference, creative ability, and leadership are the same as in Experiment 1.

#### 3.1.4. Experimental Procedure

The leader election task was added before the group creativity task, and the other procedure was the same as in Experiment 1. At first, the participants filled out their personal information and complete the pre-test assessment including the interpersonal communication ability, extraverted personality, cooperative preference, and creative ability. Next, three members recommended a leader through the leader election task. Then, the three members collaborated to complete the “Product Improvement Task” which is the same as Experiment 1. At last, all members completed the assessment of the leadership and rated the difficulty of the group task.

In the leader election task, three members conducted a discussion within 5–10 min focusing on the open-ended question “What are the characteristics and responsibilities of the leader?”. After the discussion, members selected the leader through self-nomination or nomination based on the performance during the discussion. After the leadership roles were determined, we reinforced each member’s role by asking the leader if he or she accepted his or her leader’s identity and asking the other two members if they approved of the elected leader.

#### 3.1.5. Data Coding

The data coders and coding rules in Experiment 2 were the same as in Experiment 1.

### 3.2. Results

#### 3.2.1. Individual Differences between Roles

Before analyzing the dynamic behaviors of elected leaders and their relationship to group performance, we tested whether the individual characteristics differed across roles. A series of repeated measures ANOVA analysis of variances were used to compare the interpersonal communication ability, extraverted personality, cooperative preference, and creative ability of different roles within the group. The results showed that there were significant differences in extraverted personality between roles (F (2, 29) = 4.76, *p* = 0.012, η^2^_partial_ = 0.14, 95%CI [0.01, 0.44]), and post-hoc multiple comparisons found that the extraversion of the leader (M = 2.73, SD = 0.17) was significantly higher than that of follower 2 (M = 2.13, SD = 0.18, *p* = 0.007), while there was no difference with that of follower 1 (M = 2.84, SD = 0.20, *p* = 0.675); the extraversion of follower 1 was significantly higher than that of follower 2 (*p* = 0.015). However, the repeated measures ANONA analysis about the interpersonal communication ability, cooperative preference, and creative ability showed that there were no differences between roles (interpersonal communication ability: F (2, 29) = 0.23, *p* = 0.798; cooperative preference: F (2, 29) = 0.08, *p* = 0.927, creative ability: F (2, 29) = 0.64, *p* = 0.533). We also compared the task difficulty assessed by members using repeated measures ANOVA analysis, and the result showed that the difference between roles was marginally significant (F (2, 29) = 2.89, *p* = 0.064, η^2^_partial_ = 0.09). Post-hoc multiple comparisons found that the difficulty assessed by follower 2 (M = 1.90, SD = 0.88) was significantly higher than that of follower 1 (M = 1.40, SD = 0.67, *p* = 0.023), and there was no difference between the difficulty assessed by the leader (M = 1.60, SD = 0.72) and the two followers (*p* > 0.05).

#### 3.2.2. Behavioral Characteristics of the Elected Leader in the Processes of Group Creative Problem Solving

**Figure 3:**
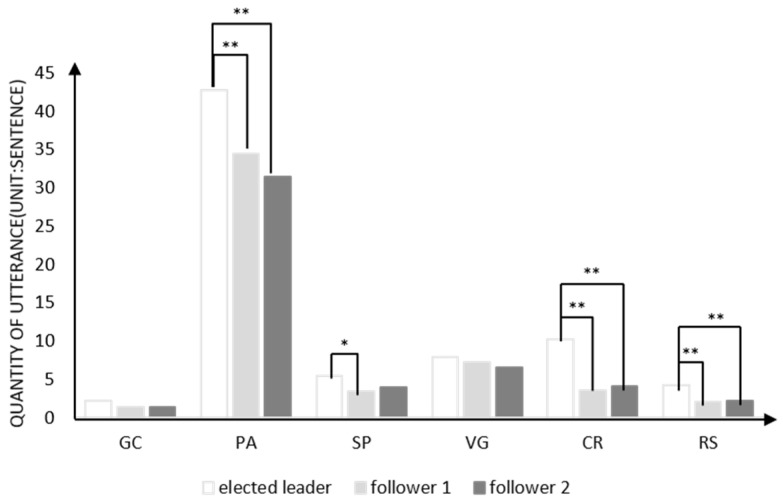
Problem-solving performance of elected leader and followers in group creativity. Note: *: *p* < 0.05, **: *p* < 0.001.

A series of repeated measures ANOVA analyses were used to compare differences in the quantity of different roles’ utterances during creative problem solving in order to explore the problem-solving behavior of elected leaders. The results revealed that there were significant differences in the total quantity of utterances during problem solving between the leader, follower 1, and follower 2 (F (2, 29) = 18.11, *p* < 0.001, η^2^_partial_ = 0.38, 95%CI [0.26, 0.69]), post-hoc multiple comparisons found that the total quantity of leader’s utterances (M = 72.77, SD = 20.62) was more than that of follower 1 (M = 52.00, SD = 16.20, *p* < 0.001) and follower 2 (M = 49.50, SD = 15.74, *p* < 0.001), while there was no difference between follower 1 and follower 2. As shown in Figure 3, the results of different utterances about problem solving between roles were different from Experiment 1. For the quantity of utterance about goal clarification and viewpoint generation, there were no differences between roles (GC: F (2, 29) = 2.92, *p* = 0.062; VG: F (2, 29) = 1.38, *p* = 0.260). For the quantity of utterances about problem analysis, there were significant differences between roles (F (2, 29) = 11.27, *p* < 0.001, η^2^_partial_ = 0.28, 95%CI [0.14, 0.60]), post-hoc multiple comparisons found that the quantity of utterances about problem analysis of the leader (M = 42.77, SD = 13.13) was more than that of follower 1 (M = 34.40, SD = 11.93, *p* = 0.001) and follower 2 (M = 31.40, SD = 12.62, *p* < 0.001), while there was no difference between follower 1 and follower 2 (*p* = 0.271). For the quantity of utterances about strategy planning, there were significant differences between roles (F (2, 29) = 3.43, *p* = 0.039, η^2^_partial_ = 0.11, 95%CI [0.00, 0.39]), post-hoc multiple comparisons found that the quantity of utterances about strategy planning of the leader (M = 5.43, SD = 3.40) was more than that of follower 1 (M = 3.37, SD = 2.58, *p* = 0.012), but there was no significant difference with follower 2 (M = 4.00, SD = 3.56, *p* = 0.111); there was also no difference between follower 1 and follower 2 (*p* = 0.422). For the quantity of utterances about control and reflection, there were significant differences between roles (F (2, 29) = 21.71, *p* < 0.001, η^2^_partial_ = 0.43, 95%CI [0.32, 0.72]). Post-hoc multiple comparisons found that the quantity of utterances about control and reflection of the leader (M = 10.20, SD = 6.39) was more than that of follower 1 (M = 3.53, SD = 2.92, *p* < 0.001) and follower 2 (M = 4.03, SD = 3.73, *p* < 0.001), but there was no difference between the two followers (*p* = 0.563). For the quantity of utterances about retrospective summary, there were significant differences between roles (F (2, 29) = 9.58, *p* < 0.001, η^2^_partial_ = 0.25, 95%CI [0.10, 0.57]), post-hoc multiple comparisons found that the quantity of utterances about retrospective summary of the leader (M = 4.27, SD = 3.88) was more than that of follower 1 (M = 2.03, SD = 2.41, *p* < 0.001) and follower 2 (M = 2.17, SD = 2.51, *p* = 0.002), but there was no difference between the two followers (*p* = 0.796).

In addition, we also calculated Pearson correlation coefficients between the quantity of different utterances of emergent leaders and every indicator of group creativity to reveal the role of emergent leaders in group creative performance. The results are shown in Table 4, the total quantity of leader utterances was neither correlated with novelty nor appropriateness (*p* > 0.05). Meanwhile, the quantity of leaders’ utterances about viewpoint generation was both positively correlated with the novelty (*r* = 0.38, *p* = 0.039) and appropriateness (*r* = 0.38, *p* = 0.040) significantly, while there was no significant correlation between the quantity of utterances about goal clarification, problem analysis, strategy planning, control and reflection, retrospective summary, and every indicator of group creative performance (*p* > 0.05).

#### 3.2.3. The Characteristics of Turn-Taking of Elected Leaders in Interpersonal Interactions

Consistent with Experiment 1, the ratio of the quantity of turns in each conversation to the total quantity of turns was used as the frequency of turns in different conversation directions for the subsequent analysis, considering the difference in the total quantity of turns in each group. Repeated measures ANOVA analysis was used to compare the differences in the total frequency of turns between roles, while a series of paired samples *t*-tests were used to compare the differences in the frequency of turns between the leader and the follower in different conversations.

Repeated measures ANOVA analysis revealed that there was no significant difference in the total frequency of turns among roles (F (2, 29) = 1.69, *p* = 0.193). Paired-samples *t*-test was used to compare the differences in the frequency of turns between L←F_1_ and F_1_←L, L←F_2_ and F_2_←L, L←F_1_ and L←F_2_, F_1_←L and F_2_←L, respectively. We found that the frequency of turns of L←F_1_ (M = 0.19, SD = 0.07) was higher than F_1_←L (M = 0.17, SD = 0.08) (t (29) = 2.54, *p* = 0.017, Cohen’ s d = 0.46, 95%CI [0.08, 0.84]); the frequency of turns of L←F_2_ (M = 0.17, SD = 0.07) was lower than F_2_←L (M = 0.18, SD = 0.08) (t (29) = −2.33, *p* = 0.027, Cohen’s d = −0.43, 95%CI [−0.80, −0.05]). There was no difference between the frequency of turns of L←F_1_ and L←F_2_ and between F_1_←L and F_2_←L (L←F_1_ and L←F_2_: t (29) = 0.98, *p* = 0.336; F_1_←L and F_2_←L: t (9) = −0.05, *p* = 0.960).

In addition, we also calculated the Pearson correlation coefficients between the quantity of L←F_1_, L←F_2_, F_1_←L, F_2_←L, and every indicator of group creativity. The results are shown in Table 5, the frequency of the turns of L←F_1_ and L←F_2_ was neither correlated with the appropriateness nor novelty of group creativity (*p* > 0.05), while the frequency of turns of F_1_←L was positively correlated with the appropriateness significantly (*r*_F1←L_ = 0.41, *p* = 0.025).

### 3.3. Discussion

Through free discussion of an open-ended question, the members of the group get to know each other briefly, and then members elected the group’s leader based on their performance during the discussion. In the formal experiment, the elected leader and the follower collaborated on the group creativity task. By analyzing the dynamic behaviors of elected leaders in problem solving and their relationship with group creativity performance during the discussion, the mechanism of elected leadership in group creativity was revealed.

By comparing the differences between the interpersonal communication ability, extroverted personality, and cooperative preferences among the three roles, we found that elected leaders scored significantly higher on extroversion than follower 2, while there were no differences in other variances among the three roles. Extroverted individuals enjoy socializing, especially in large groups, and are more assertive and talkative. Previous research on personality traits in leaders also found a positive relationship between extroverted personality traits and leadership (Conard 2020). These results also provide some evidence of the validity of the role classification in this study.

Meanwhile, in this experiment, by comparing the quantity of utterances of elected leaders and followers, we found that the total quantity of utterances of elected leaders was more than that of the followers, as well as the quantity of utterances about problem analysis, strategy planning, control and reflection, and retrospective summary, while only the quantity of utterances about viewpoint generation was positively related to both novelty and appropriateness of group creativity. At the same time, by comparing the frequency of turns of elected leaders and followers during discussions, we found that elected leader has no difference with two followers in terms of the total frequency of turns, while the frequency of turns of elected leaders was significantly higher than that of follower 1 in the conversation with follower 1, while the opposite result was shown in the conversation with follower 2. There was no relationship between the frequency of turns of the elected leader and group creative performance.

Combining the results of Experiment 1, we found that the functional mechanisms in group creativity were not consistent across different leaders. Compared to emergent leaders who engage more in interpersonal interactions, elected leaders focus more on problem solving. Through the two experiments, the behavioral performance of emergent and elected leaders and their effects on group creativity were revealed from the perspective of dynamics.

## 4. Discussion

In this study, we explored the role of leaders in group creativity by analyzing the utterance content related to problem solving and the sequence of turn-taking in interpersonal interactions of the emergent leader and the elected leader in the group creativity task. The results showed us that: (1) For the emergent leader, in terms of the quantity of utterance related to the problem solving, the quantity of utterances about goal clarification, problem analysis, strategy planning, viewpoint generation, control and reflection, and retrospective summary of the emergent leader were all not different from that of the two followers, and the quantity of utterances about retrospective summary of the emergent leader was positively related to the appropriateness of the group creativity. At the same time, in terms of the frequency of turns, the total frequency of turns of the emergent leader was significantly higher than that of the followers, and the conversations between the emergent leader and follower were not counterbalanced in which the frequency of turns of the emergent leader was higher than that of the follower in both conversations. We also found that the frequency of turns of the emergent leader was positively related to the appropriateness of the group creativity significantly. (2) For the elected leader, in terms of the quantity of utterance related to the problem solving, the quantity of utterances about problem analysis, strategy planning, control and reflection, and retrospective summary of the elected leader were all more than that of the two followers, and the quantity of utterances about viewpoint generation of elected leader was positively related to both novelty and appropriateness of the group creativity. At the same time, in terms of the frequency of turns, the total frequency of turns of the elected leader was the same as that of the two followers, and the conversations between elected leaders and followers were not counterbalanced in which the frequency of turns of the elected leader was higher than that of follower 1 while an opposite result was observed in the conversation between the elected leader and follower 2. We also found that the frequency of turns of the elected leader was neither related to the novelty nor appropriateness of the group creativity.

### 4.1. Behavioral Characteristics of Leaders in Group Creativity during Problem Solving

Everyone is creative, group creativity is the result of members thinking together to create (Leonard-Barton and Swap 1999). Members of the group use internal cues to search for relevant information from long-term memory based on their own knowledge system to form new ideas, then share them with other members. At the same time, through interpersonal interaction, other members’ ideas can also serve as external cues to prompt individuals to generate more ideas, acting as a facilitator of thinking (Paulus and Brown 2007). The leader, as an important role of the group, has multiple influences on group creativity. At first, as one member of the group, the leader is equally expected to generate novel and appropriate ideas or solutions as other members (Gerpott et al. 2019). More importantly, the leader, as the core member of the group, is expected to have high level of creative thinking abilities, and thus needs to take responsibility for leading the thinking of others (Mumford and Licuanan 2004).

We found that the emergent leader did not differ significantly from followers in the quantity of utterances about problem solving in group creativity in Experiment 1. This suggests that the leader and followers performed consistently during creative problem solving because there were no significant differences in creative abilities among them. However, in the subsequent correlational analysis, we found that the quantity of utterances about retrospective summary of the emergent leader was associated with the appropriateness of group creativity. It could be reasoned that the emergent leader has a greater tendency to be appropriate in reviewing and summarizing prior perspectives and facilitate consensus among members. Previous studies related to leadership have found that in addition to their own superior knowledge and competence, leaders’ interpersonal relationships with their members are also important factors (Eva et al. 2019; Zhang et al. 2015). Meanwhile, some studies have shown that the expression of creative or novel ideas may diminish judgments of leadership potential (Mueller et al. 2011), because individuals who express creative ideas are often perceived as mavericks, which is also contradictory to the characteristics (concurrent, collective, collaborative, and compassionate) of leaderful managers (Raelin 2005). That means, the emergent leader emphasizes the appropriateness rather than the novelty of group goals by reviewing and summarizing existing ideas in the discussion with members, and ultimately influences the group performance.

For the elected leader, we found that the quantity of utterances including problem analysis, strategy planning, control and reflection, and retrospective summary of elected leaders were more than that of followers. This is consistent with the research related to knowledge leadership in which leaders need to act as role models, orienting learning, and supporting the learning process at the group and individual levels (Viitala 2004). Furthermore, we also found that the quantity of utterances about viewpoint generation of the elected leader was positively related to both novelty and appropriateness of group creativity. This result was consistent with the role theory, which proposed that once a leader’s social role is clarified, he or she assumed more responsibility in the group and is accountable for the group’s ultimate performance than other members (Van Lange et al. 2011). As an important role in the group, the leader plays a more direct role in being actively engaged in the production and refinement of new ideas (Mumford et al. 2003). During the process of solution/alternatives generation, leaders can influence the results by providing instruction to the followers or subordinates, including not only techniques to facilitate idea generation but also what the goals of the specific process should be (Reiter-Palmon and Illies 2004). Therefore, compared to followers, elected leaders pay more attention to the achievement of group goals, and generate more novel and appropriate ideas in the process of problem solving to lead the way of thinking, promote the discussion among members and make all the members reach a consensus.

### 4.2. Turn-Taking of Leaders in Group Creativity during Interpersonal Interaction

In the process of group discussion, members are interdependent on each other and convert inputs to outcomes through cognitive, verbal, and behavioral activities directed toward organizing taskwork to achieve collective goals (Marks et al. 2001). The leader, as an important member of the group, the effectiveness of his role in the group occurs through the process of his interaction with other members (Van Knippenberg and Hogg 2003). As verbal activities are the most obvious form of interpersonal interaction, analyzing turn-taking can reveal the attitudes of individuals (Ravenet et al. 2015). Thus, the mechanism of the leader role in group creativity can be explained to some extent by analyzing the leader and member’s turn-taking in problem solving.

In the analysis of the turn-taking in the conversation, we found that the frequency of turns of the emergent leader was more than the two followers during the problem solving. Research on the relationship between conversation and emerging leadership showed that the stronger leadership the individuals show, the more utterances they initiate and receive (Misiolek and Heckman 2005). Wickham and Walther (2007) also suggested that the member who is perceived as the most frequent communicator tends to be perceived as an emergent leader in leaderless group discussion. Another study on emergent leadership showed that extroverted personality, as well as communication ability, can predict leader identity in leaderless group discussion (Scymcyk 2020). The results of this study also found that emergent leaders have higher communication ability and played the leadership role through responsive interaction in conversation with other members, ultimately achieving group effectiveness.

While the “babble hypothesis” of emergent leadership suggests that the quantity of utterances, not its quality, determines leader emergence, and the quantity of utterances reflects the degree of individual involvement in group activities (Adams 2009; Hawkins 1995; MacLaren et al. 2020), these results are not consistent with this study. One possible reason for this is that communication ability and competence are more important than communication frequency in small groups (Jiang et al. 2015). In addition, compared to the quantity of utterances, the frequency of turns of individuals can also be an index of participation. In the study by (Badura et al. 2018), he suggests that greater participation may lead group members to conclude that active participators have more expertise and knowledge of group tasks, and are more motivated and interested in performing leadership roles, thus the participation may signal to others not only an ability but also a willingness to contribute to the group.

In Experiment 2, for the elected leaders, we found that there was no difference in the total frequency of turns between the elected leader and followers. It is generally believed that when a leader with a clear identity communicates to other members within the group, due to the unequal social status of the two, there may be differences in initiated and received conversation among different roles. However, in the present study, on one hand, the group was formed temporarily and would be dissolved after the experiment. On the other hand, the leader was approved by followers because the leader was freely elected by them (Elgie et al. 1988). Therefore, there was less pressure on the leader to demonstrate his leadership through responsive interaction during the creative problem solving. At the same time, the discussion is more focused on creative problem solving, and thus the behavior of elected leaders is more task-oriented and takes more responsibility for task goal achievement. As a result, the elected leader would pay more attention to problem solving rather than to interpersonal interaction, as evidenced by no significant difference between the frequency of turns of elected leaders and followers.

Interestingly, we distinguished the two followers into follower 1 and follower 2 based on the self-assessment of their leadership. By comparing the frequency of turns between the leader and the two different followers, it was found that the emergent leader took turns more than follower 2 in the leaderless group discussion, while the opposite result was observed in the conversation between the elected leader and follower 2. One of the possible reasons for this result is that in the group with a leader, individuals with low leadership may have greater follower characteristics and may have more responsive interactions with the leader. In the study by (Zhu et al. 2009), he found that follower characteristics have a positive relationship with follower work engagement at the individual level. However, the relationship between follower characteristics and leadership effectiveness is complex (Matthews et al. 2021), and the explanation for this result remains to be explored in depth.

As for the relationship between leaders’ interpersonal interaction and group performance, the findings suggested that the frequency of turns of the emergent leader was positively related to the appropriateness of the group creativity, and the frequency of turns of the elected leader has no relationship with the appropriateness and novelty of the group creativity. Previous research on leaders suggests that when individuals perceive themselves as leader, they will strengthen their responsibility as leaders and further develop their leadership skills (Day et al. 2009). In our study, the responsibility of the elected leader was reinforced through the leader election task. Basadur et al. (2000) found that creative problem solving can enhance individuals’ attitudes toward active divergence and the tendency to avoid premature convergence. This attitude will eventually lead to focus on problem solving rather than on interpersonal interaction during the process of creative problem solving. Moreover, knowledge teams pay more attention to problem solving, so the leaders in these groups are more responsible for generating viewpoints and leading the thinking of members, rather than for interpersonal interaction. Therefore, the group performance has a positive relationship with the quantity of utterances about problem solving of the elected leader, rather than the frequency of turns. In other words, when the role of the leader was identified, although the total quantity of utterances increased, the interpersonal interaction with followers was decreased conversely, because the leader perceived more responsibility as the problem solver. In contrast, when the role of the leader was not identified, the leader was more like a participant, and engaged in the discussion by interacting with other members. Therefore, the group performance has a positive relationship with his responsive interactions.

## 5. Conclusions

Overall, this study explored dynamic behaviors including the utterances and turn-taking of the emergent leader and the elected leader during group creative problem solving from the perspective of group interaction processes through two experiments, and analyzed the possible mechanisms of leaders’ roles in group creativity. In leaderless group discussions, leaders did not express their personal viewpoints prominently and took more turns in interpersonal interactions, thus their frequency of turns were related to group creativity, while when the role of leader was identified, leaders perceived more responsibility and expressed more viewpoints, thus the quantity of utterances about problem-solving of elected leaders was related to group creativity.

## Figures and Tables

**Table 1:** The meaning and examples of utterance categories.

Category	Meaning	Example
GC	The discussions related to task objectives	“This idea is not novel”
PA	Including the analysis of the problem and the analysis of the solution	“Many existing umbrellas are not easy to store”
SP	Mainly involves a description of problem-solving strategies	“Let’s think about it from the perspective of appearance design”
VG	Specific methods and ideas for problem solving or program improvement	“Reflective strips can be added to the umbrella surface”
CR	Control and reflection on the discussion process, mainly on the interactive process	“You say first”
RS	Review of existing ideas	“We have talked about adding reflective strips to the umbrella surface, adding GPS, adding flashlights…”

Notes: GC: goal clarification; PA: problem analysis; SP: strategy planning; VG: viewpoint generation; CR: control and reflection; RS: retrospective summary.

**Table 2:** Correlation between the quantity of utterances of emergent leader and the indicators of group creativity.

	M	SD	Total	GC	PA	SP	VG	CR	RS
novelty	3.81	0.98	0.161	−0.302	0.050	0.341	0.149	0.326	0.334
appropriateness	3.93	0.72	−0.089	−0.155	−0.289	0.157	0.114	0.298	0.380 *

Note: *: *p* < 0.05.

**Table 3:** Correlation between the frequency of turns of the emergent leader and the indicators of group creativity.

	M	SD	Total	L←F_1_	L←F_2_	F_1_←L	F_2_←L
novelty	3.81	0.98	0.302	0.310	0.289	−0.001	−0.327
appropriateness	3.93	0.72	0.392 *	0.369 *	0.407 *	−0.140	−0.310

Note: *: *p* < 0.05.

**Table 4:** Correlation between the quantity of utterances of the elected leader and the indicators of group creativity.

	M	SD	Total	GC	PA	SP	VG	CR	RS
novelty	4.52	0.86	0.210	−0.063	0.087	0.265	0.379 *	0.127	0.043
appropriateness	4.70	0.64	0.156	−0.197	0.066	0.054	0.377 *	0.077	0.148

Note: *: *p* < 0.05.

**Table 5:** Correlation between the frequency of turns of the elected leader and the indicators of group creativity.

	M	SD	Total	L←F_1_	L←F_2_	F_1_←L	F_2_←L
novelty	4.52	0.86	−0.027	−0.039	0.010	0.056	0.044
appropriateness	4.70	0.64	0.082	0.167	−0.076	0.409 *	−0.158

Note: *: *p* < 0.05.

## Data Availability

The data presented in this study are available on request from the corresponding author. The data are not publicly available due to privacy.
